# Eurasian Avian-like M1 Plays More Important Role than M2 in Pathogenicity of 2009 Pandemic H1N1 Influenza Virus in Mice

**DOI:** 10.3390/v13122335

**Published:** 2021-11-23

**Authors:** Lixiang Xie, Guanlong Xu, Lingxiang Xin, Zhaofei Wang, Rujuan Wu, Mingqing Wu, Yuqiang Cheng, Hengan Wang, Yaxian Yan, Jingjiao Ma, Jianhe Sun

**Affiliations:** 1 School of Agriculture and Biology, Shanghai Jiao Tong University, Shanghai 200240, China; xielixiang2020@gmail.com (L.X.); wzfxlzjx@sjtu.edu.cn (Z.W.); wrujuan9425@163.com (R.W.); shixianggg0@163.com (M.W.); 1987lccyq@163.com (Y.C.); hawang@sjtu.edu.cn (H.W.); yanyaxian@sjtu.edu.cn (Y.Y.); 2 China Institute of Veterinary Drug Control, Beijing 100081, China; xuguanlongw@163.com (G.X.); xinxeya@sina.com (L.X.); 3 Shanghai Key Laboratory of Veterinary Biotechnology, Shanghai 200240, China

**Keywords:** influenza, pathogenicity, 2009 H1N1 pandemic, M1, M2, Eurasian avian-like

## Abstract

Reassortant variant viruses generated between 2009 H1N1 pandemic influenza virus [A(H1N1)pdm09] and endemic swine influenza viruses posed a potential risk to humans. Surprisingly, genetic analysis showed that almost all of these variant viruses contained the M segment from A(H1N1)pdm09, which originated from Eurasian avian-like swine influenza viruses. Studies have shown that the A(H1N1)pdm09 M gene is critical for the transmissibility and pathogenicity of the variant viruses. However, the M gene encodes two proteins, M1 and M2, and which of those plays a more important role in virus pathogenicity remains unknown. In this study, the M1 and M2 genes of A(H1N1)pdm09 were replaced with those of endemic H3N2 swine influenza virus, respectively. The chimeric viruses were rescued and evaluated *in vitro* and in mice. Both M1 and M2 of H3N2 affected the virus replication *in vitro*. In mice, the introduction of H3N2 M1 attenuated the chimeric virus, where all the mice survived from the infection, compared with the wild type virus that caused 100 % mortality. However, the chimeric virus containing H3N2 M2 was still virulent to mice, and caused 16.6% mortality, as well as similar body weight loss to the wild type virus infected group. Compared with the wild type virus, the chimeric virus containing H3N2 M1 induced lower levels of inflammatory cytokines and higher levels of anti-inflammatory cytokines, whereas the chimeric virus containing H3N2 M2 induced substantial pro-inflammatory responses, but higher levels of anti-inflammatory cytokines. The study demonstrated that Eurasian avian-like M1 played a more important role than M2 in the pathogenicity of A(H1N1)pdm09 in mice.

## 1. Introduction

A swine-origin H1N1 influenza virus, first reported in Mexico in April 2009, resulted in human infection, and became widespread. In only one month, the A(H1N1)pdm09 infection was confirmed with 180,000 cases reported globally [1]. In June 2009, the global distribution prompted the World Health Organization (WHO) to declare the first influenza pandemic of the 21st century. The influenza human infection cases of A(H1N1)pdm09 were more predominant in the 2009/2010 season than human seasonal H1N1 and H3N2 subtypes, and caused high mortality rates [2,3]. The gene analysis confirmed this virus to be a reassortant virus with six genes (PB2, PB1, PA, HA, NP, and NS) from North American triple-reassortant influenza viruses, and two genes (M, NA) from Eurasian avian-like swine influenza viruses [4]. The disease patterns of A(H1N1)pdm09 infection cases were markedly different from those seen during seasonal influenza infection, in that many of the patients were young people [5]. In addition, the A(H1N1)pdm09 jumped from humans to different animal species, including dogs, turkeys, cats, pigs, ferrets, and other wildlife [6,7,8,9,10]. Pigs are considered to be “mixing vessels” because they have receptors for both avian- and human-like influenza viruses. The introduction of A(H1N1)pdm09 into pigs was noticed in several countries, and they had reassortment events with endemic swine influenza viruses (SIVs) [9,11,12]. Influenza A H3N2 variant viruses (H3N2v) possessing the M gene derived from A(H1N1)pdm09 were initially detected in pigs of United States in 2010 [13]. Then, these viruses infected humans, and caused deaths with limited human-to-human transmission in 2011 [14,15]. Triple-reassortant SIVs have been circulating in pigs since 1998, causing only sporadic pig-to-human transmission in the United States [16,17]. Many studies identified that almost all the variant viruses contained the M gene from A(H1N1)pdm09 [18,19]. The Eurasian origin M segment has been confirmed to contribute to the transmissibility, aerosol release, and morphology of the A(H1N1)pdm09 virus [20]. Chou Y. et al. has reported that the M gene of A(H1N1)pdm09 enhanced the transmissibility of H3N2v viruses [21].

Matrix protein 1 (M1) and matrix protein 2 (M2) are expressed by the M gene of the influenza virus [22]. M1 is encoded by unspliced mRNA1, and M2 protein is encoded by spliced mRNA2 through alternative RNA splicing. M1 and M2 proteins are involved in many steps during viral replication, including entry, uncoating, assembly, as well as budding. M1 is a structural protein that forms the inner structure of the virion, and is associated with virion morphology [23,24,25]. At early stages of infection, M2 tetramer acts as a highly selective proton-conducting channel during virus uncoating. The low pH < 6 and influx of K(+) ions trigger a conformational change of M1 and stability of ribonucleoprotein (RNP) complexes [26,27], which are required to release the viral genome to the cytoplasm [28]. At later stages of infection, M1 and M2 proteins play important roles in virus assembly and budding [29], which are crucial for virus survival and pathogenicity. Upon influenza virus assembly, M1 recruits newly synthesized vRNP to budding sites for genome packaging. M2 is recruited to the peripheral of the lipid raft, and stabilizes the site of budding [29,30]. M1 is the most abundant protein and the major force to mediate the membrane curvature [31]. Later in the budding process, M2 localizes to the boundary of the lipid raft, and alters the membrane scission by inducing negative Gaussian curvature (NGC), mediating the endosomal sorting complex required for transport (ESCRT)-independent membrane scission at the neck of the budding virion, which plays an important role in the final step of morphogenesis [32,33].

The M gene contributes to the transmissibility and pathogenicity of the A(H1N1)pdm09 virus. However, the influence of M1 and M2 on the pathogenicity of the A(H1N1)pdm09 virus remains unclear. A(H1N1)pdm09 adapts to mice very well, and could cause mouse morbidity and mortality. The infected mouse will show clinical signs, including ruffled fur, loss of appetite, weight loss, and hunched posture. However, the mouse will not exhibit the typical symptoms of influenza to humans. Herein, to explore the role of M1 and M2 protein in viral pathogenicity of A(H1N1)pdm09, chimeric viruses containing either the M1 or M2 gene from the H3N2 subtype endemic swine influenza virus in the background of A(H1N1)pdm09 were rescued and evaluated *in vitro*, and in mice as a mammalian model [34,35]. The H3N2 M1 and H3N2 M2 proteins affected the virus replication *in vitro*. Introduction of either H3N2 M1 or H3N2 M2 attenuated the chimeric viruses compared with the wild type virus. The chimeric virus encoding H3N2 M1 did not cause mouse deaths, but caused lower inflammatory cytokine and higher anti-inflammatory cytokine levels than the wild type virus. The chimeric virus encoding H3N2 M2 caused 16.7% mortality in mice, and a similar body weight loss to the wild type virus infected group. All the results suggested that the Eurasian avian-like M1 played a more important role than M2 in the virulence of A(H1N1)pdm09 in mice.

## 2. Materials and Methods

### 2.1. Cells and Viruses

Madin-Darby canine kidney (MDCK) and human embryonic kidney (HEK) 293T cells were maintained in Dulbecco’s modified Eagle medium (DMEM; Gibco, Grand Island, NY, USA), supplemented with 10% fetal bovine serum (FBS; Gibco, Grand Island, NY, USA), 1×L-glutamine (100×; Gibco, Grand Island, NY, USA), 1×NEAA (100×; Gibco, Grand Island, NY, USA), and 1×Antibiotic-Antimycotic (100×; Gibco, Grand Island, NY, USA) at 37 °C with 5% CO_2_.

A/California/04/2009 (H1N1) (CA09) is a representative strain of the 2009 H1N1 pandemic virus rescued by reverse genetics with standard procedures, as previously described [36]. A/Swine/Shandong/R3/2005 (H3N2) is an endemic SIV maintained in our laboratory. The rescued viruses were propagated and titrated on MDCK cells.

### 2.2. Construction of Plasmids

**Figure 1:**
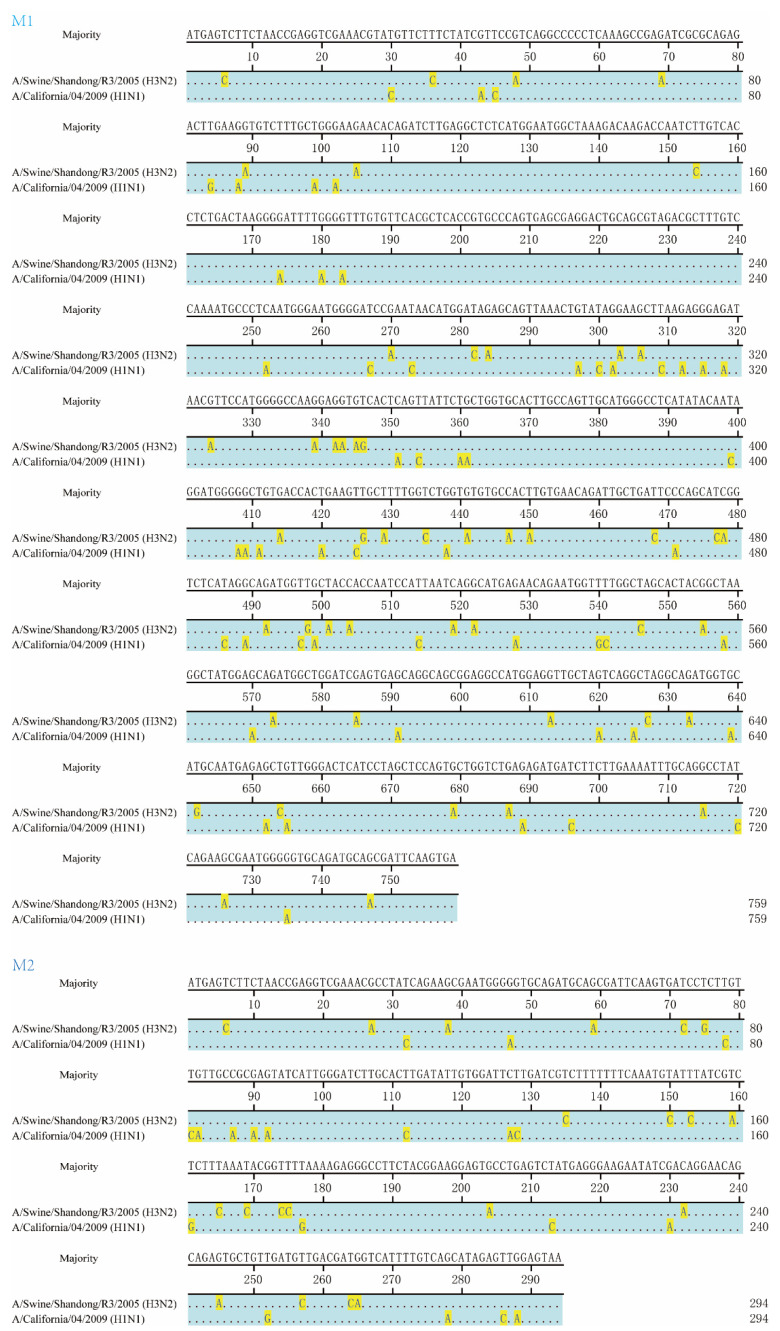
Alignment of M1 and M2 genes between A/Swine/Shandong/R3/2005 (H3N2) and A/California/04/2009 (H1N1) strains. Alignment was performed using MegAlign in Lasergene version 7.1.0 (DNASTAR, Madison, WI). The majority sequence is shown in the top row, the same nucleotides are hidden, and the different nucleotides are highlighted.

**Figure 2:**
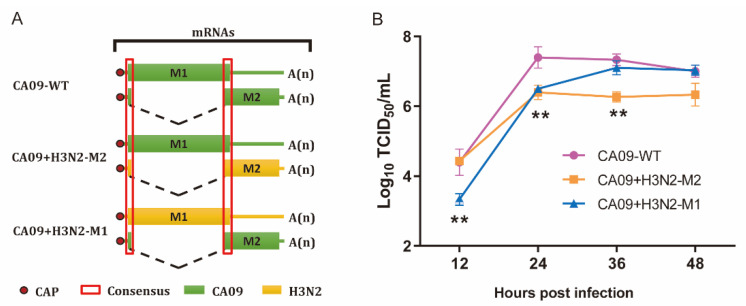
Influence of the M1 and M2 gene exchange to CA09-WT. (**A**) Schematic diagram of chimeric viruses. The CA09 M gene was replaced with CA09-M1 + H3N2-M2 and H3N2-M1 + CA09-M2, amplified by overlapping PCR without changing the original amino acid sequences. The overlapping segments of M1 and M2 are consensus between CA09 and H3N2. CA09-WT and chimeric virus CA09 + H3N2-M2 and CA09 + H3N2-M1 were rescued by reverse genetic system. (**B**) Viral growth kinetics on MDCK cells using 0.001 MOI. Viruses replicated efficiently on MDCK cells. Supernatants were collected at 12, 24, 36, and 48 hpi in triplicate independently. Each plot is shown as Mean ± SEM. The experiment has been repeated at least three times, ** *p* < 0.01.

To generate chimeric M segments, first, the amino acid and gene sequences of two M sequences were aligned, and the different sites are highlighted in Figure 1. The amino acid sequence homology of M1 and M2 of CA09 and H3N2 are 92.1% and 84.5%, respectively. The amino acid sequences of overlapping segments of M1 and M2 are the same. Then, primers were designed to amplify M1 and M2 fragments of two viruses, respectively. The M1 and M2 segments were connected by overlapping PCR using universal M1 forward and M2 reverse primers [37] (Figure 2A). Primers used in the PCR are listed in Table 1. The chimeric M genes were cloned to the pHW2000 vector, and named pHW2000-CA09-M1 + H3N2-M2 and pHW2000-H3N2-M1 + CA09-M2.

### 2.3. Rescue of Recombinant Influenza a Viruses

Three viruses were rescued using a reverse genetic system: one wild type virus (CA09-WT) and two chimeric viruses containing the chimeric M segment (CA09-M1 + H3N2-M2, H3N2-M1 + CA09-M2) in the background of CA09. Briefly, 293T cells were transfected with an eight-plasmid reverse genetic system based on the pHW2000 vector. Seven plasmids, except for the M gene of CA09, combined with either intact CA09 M, CA09-M1 + H3N2-M2, or H3N2-M1 + CA09-M2, were transfected to 293T cells in 1 mL Opti-MEM (Gibco, Grand Island, NY, USA) in a 6-well-plate, respectively. One day after transfection, another 1 mL of medium containing TPCK-trypsin (Worthington, Lakewood, NJ, USA) was supplemented into the system. Two days after transfection, the supernatant was collected, and infected MDCK cells were supplemented with TPCK-trypsin. The rescued viruses were confirmed by sequencing.

### 2.4. Virus Growth Kinetic Assay

To determine the growth kinetics of the viruses *in vitro*, MDCK cells cultured in 12-well plates were infected at a multiplicity of infection (MOI) of 0.001 with the three viruses, respectively. The plaque forming unit (pfu) was converted from TCID_50_ (pfu/mL = 0.69 × TCID_5__0_/_mL_). Samples in triplicate at each time point were inoculated at the same time. The supernatant of each sample was collected at 12, 24, 36, and 48 hours post inoculation (hpi). Viral titers were determined by 50% tissue culture infectious dose (TCID_50_) analysis on MDCK cells cultured in a 96-well-plate using the Reed–Muench method.

### 2.5. Animal Study

To evaluate the pathogenicity of the viruses *in vivo*, 48 four-to-six-week-old female SPF BALB/c mice were randomly allocated into four groups (three infection groups and one control group), and each group contained twelve mice. Mice in three infection groups were inoculated intranasally with the 10^5.5^ TCID_50_ designated virus under general anesthesia with Zoletil ™ (VIRBAC, Carros, France). The control mice were inoculated intranasally with 50 μL phosphate-saline buffer (PBS; Gibco, Grand Island, NY, USA). Mice were weighed and monitored daily for clinical signs of infection for 14 days, such as ruffled fur, anorexia, and dyspnea. The criteria for the humane endpoint of mice is greater than 25% weight loss [38]. For each group, three mice were euthanized on 3 and 5 dpi. During necropsy, halves of the mouse lung were collected and placed in DMEM containing 1× Antibiotics-Antimycotic (100×; Gibco, Grand Island, NY, USA), and stored at −80 °C for virus titration. The mouse lungs were homogenized by weight/volume = 1:10 and centrifuged to collect the supernatant. The viral loads in the mouse lungs were titrated on MDCK cells in a 96-well-plate [39]. The other halves of the mouse lung of 5 dpi were fixed in 4% paraformaldehyde, and stained with haematoxylin and eosin (H&E) for histopathological evaluation. The influenza viral antigens in lungs of 5 dpi were detected with anti-H1N1 NP rabbit polyclonal antibody (GenScript, Nanjing, China) by an immunohistochemistry (IHC) assay. The slides were systemically scanned by microscopy. Evaluation of the histopathological lesion of lung sections were performed by a pathologist in a blinded fashion. The lesions were scored with 0–4 to assess the lung damage affected by interstitial inflammation, peribronchial inflammation, bronchial luminal exudate, perivascular infiltrate, and parenchymal pneumonia according to the following criteria: (0) no significant lesions; (1) up to 30%; (2) up to 50%; (3) up to 70%; (4) 70% and more.

### 2.6. Determination of Cytokine Levels in Mouse Lungs

Real-time quantitative RT-PCR was employed to determine the cytokine levels in mouse lungs, such as interleukin (IL)-1β, IL-10, interferon (IFN)-β, and tumor necrosis factor (TNF)-α. The glyceraldehyde-3-phosphate dehydrogenase (GAPDH) gene was used as a control housekeeping gene. Briefly, the lung samples were obtained during the necropsy, and stored at −80 °C. PBS control on day 3 was used as the reference point (=1) of day 3 samples, and PBS on day 5 was for day 5 samples. The total RNA was isolated from lung tissues using TRIzol^®^ LS Reagen (Invitrogen™, Carlsbad, CA, USA), and was transcripted with SuperScript^®^ III Reverse Transcriptase (Invitrogen™, Carlsbad, CA, USA) to cDNA following manufacture’s instruction. The quantitative PCR was conducted in a 20 μL reaction volume, containing 10 μL 2× ChamQ SYBR qPCR Master Mix (Low ROX Premixed; Vazyme, Nanjing, China), 0.4 μL of each primer (10 μM), 7.2 μL RNase-free water, and 2 μL cDNA. The reaction was performed on an Applied Biosystems 7500 Fast Real-Time PCR System. The real time PCR condition was set as follows: 95 °C for 30 s; then 40 cycles of 95 °C for 10 s; and 60 °C for 30 s. The primers used in the study are listed in Table 1 [40,41].

### 2.7. Ethics Statement and Statistical Analysis

The animal study was conducted in accordance with the guidelines of the Animal Care and Use Committee of Shanghai Jiao Tong University, and the animal study protocols were approved by Shanghai Jiao Tong University. The data were analyzed by analysis of variance in GraphPad prism version 5.0 (GraphPad Software Inc, La Jola, CA, USA); a *p*-value less than 0.05 was considered significant. Survival analysis was preformed using Kaplan–Meier method in R version 4.0.5 (R Foundation for Statistical Computing, Vienna, Austria).

## 3. Results

### 3.1. M1 and M2 of H3N2 Affected Replication of Chimeric Viruses In Vitro

Three viruses were generated using a reverse genetic system: wide type virus (CA09-WT), CA09 + H3N2-M1, and CA09 + H3N2-M2. The chimeric virus CA09 + H3N2-M1 contains the chimeric M gene encoding M2 protein of CA09 and M1 protein of H3N2 in the background of CA09. Similarly, the chimeric virus CA09 + H3N2-M2 harbors M1 protein of CA09 and M2 protein of H3N2. The chimeric M, HA, and NA genes of the rescued viruses were verified by sequencing, which indicated that the chimeric viruses were successfully rescued.

Viral growth kinetics was then assessed on MDCK cells. All viruses replicated efficiently on MDCK cells (Figure 2B). The CA09 + H3N2-M1 replicated to significantly lower titers at 12 hpi than the other two viruses, and significantly lower titers at 24 hpi than CA09-WT, and similar titers with the CA09-WT at 36 and 48 hpi. In contrast, the CA09 + H3N2-M2 replicated to similar titers with CA09-WT at 12 hpi, but significantly lower titers than CA09-WT at 24 and 36 hpi. In addition, the viral titers of the CA09 + H3N2-M2 were equivalent to CA09 + H3N2-M1 at 24 hpi, but statistically lower than CA09 + H3N2-M1 at 36 hpi.

### 3.2. H3N2 M1 Attenuated CA09 + H3N2-M1 in Mice

**Figure 3:**
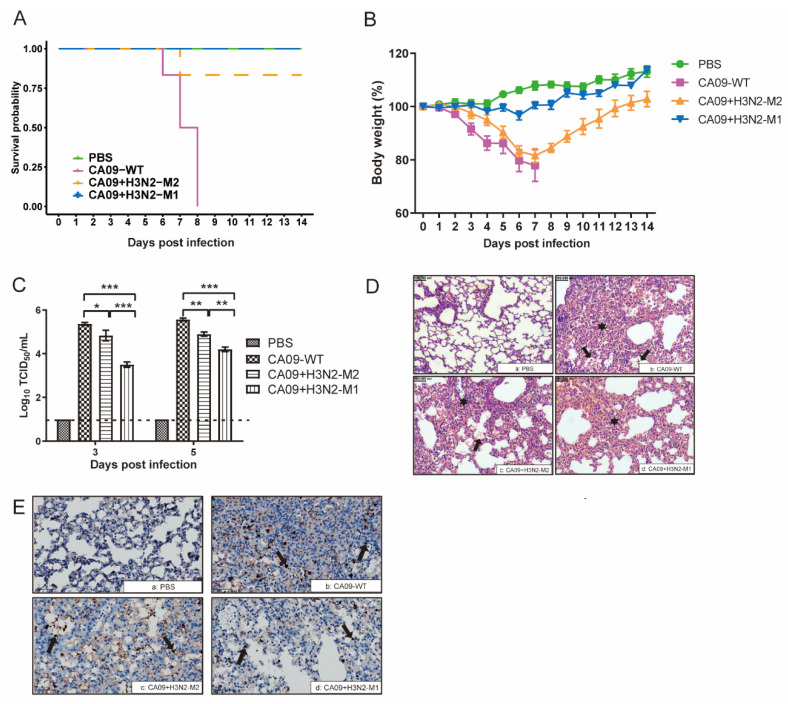
Chimeric viruses evaluated in mice. (**A**) Survival analysis was performed using the Kaplan–Meier method. The CA09-WT group showed 100% mortality, the CA09 + H3N2-M2 group showed a 16.7% mortality rate, and the mice in the CA09 + H3N2-M1 infection group survived in the study. The PBS groups showed a 100% survival rate. The criteria for the humane endpoint of mice is greater than 25% weight loss. (**B**) Body weights of mice in each group were measured daily for 14 days post infection. The CA09 + H3N2-M2 caused less body weight loss than CA09-WT, but more weight loss than the CA09 + H3N2-M1. (**C**) Virus titers in mouse lungs in infection groups on 3 and 5 dpi. The virus titers indicate Mean ± SEM in three independent experiments. Mice infected with the CA09-WT showed the highest titers, and mice infected with the CA09 + H3N2-M1 showed the lowest titers among the three groups. Significant differences were observed among the groups. The statistical analysis was performed using GraphPad Prism with two-way ANOVA, * *p* < 0.05; ** *p* < 0.01; *** *p* < 0.001. (**D**) Histopathological study of infected mouse lungs on 5 dpi. (**a**) The alveoli are clear and bronchioles are lined by normal cuboidal epithelium in the PBS group; (**b**) The alveolar and bronchiolar lumen are filled with large numbers of neutrophils, there is moderate to severe bronchiolar epithelial degeneration, and necrosis is seen in the CA09-WT group; (**c**) In the CA09 + H3N2-M2 group, some alveoli are filled with neutrophils, and there is moderate bronchiolar epithelial degeneration; (**d**) Some of the alveolar and bronchiolar lumen are filled with neutrophils in the CA09 + H3N2-M1 group. Bronchiolar and alveolar lumen infiltration is marked with asterisks, and alveoli collapse is marked with arrows. Scale bars, 100 μm. (**E**), IHC assay of mouse lung sections. Influenza antigens were detected by anti-H1N1 NP rabbit polyclonal antibody. The antigens are indicated with arrows. (**a**) PBS group; (**b**) CA09-WT group; (**c**) CA09 + H3N2-M2; (**d**) CA09 + H3N2-M1 group. Scale bars, 50 μm.

All the CA09-WT infected mice showed obvious clinical signs, such as decreased activities and ruffled fur, and the infection caused severe weight loss and 100% mortality in mice (Figure 3A,B). However, the CA09 + H3N2-M1 virus was dramatically attenuated compared with CA09-WT. The infected mice only showed slight clinical signs, and all the mice survived from the infection with only slight weight loss. The CA09 + H3N2-M1 (log_10_ TCID_50_/_mL_ = 3.5 ± 0.12) replicated to significantly lower titers in mouse lungs on 3 dpi compared with CA09 + H3N2-M2 (4.8 ± 0.24) and CA09-WT (5.4 ± 0.07), and on 5 dpi, the CA09 + H3N2-M1 (4.2 ± 0.1) infected group also showed significantly lower titer than the CA09 + H3N2-M2 (4.9 ± 0.1) and the CA09-WT (5.6 ± 0.07) infected groups (Figure 3C). All the results indicated that introduction of H3N2-M1 dramatically attenuated replication and pathogenicity of the CA09 + H3N2-M1 in mice.

The CA09 + H3N2-M2 virus caused 16.7% mortality in infected mice (Figure 3A). The mouse body weight showed that the CA09 + H3N2-M2 infected mice lost more weight than the CA09 + H3N2-M1 infected mice (Figure 3B). The CA09 + H3N2-M2 replicated efficiently in lungs with lower titers than the CA09-WT, but higher than the CA09 + H3N2-M1 infected group (Figure 3C). The CA09 + H3N2-M2 virus was still pathogenic to mice, although it was less virulent than the CA09-WT (Figure 3D). The histopathological change of lungs showed that the CA09 + H3N2-M1 caused milder bronchiolar and alveolar lumen infiltration, less alveolar collapse, and fewer lymphocytes and neutrophils observed in peribronchiolar and interstitial areas than the CA09-WT and CA09 + H3N2-M2 groups (Figure 3D). The lung lesions were scored based on pathological damage (0–4). The results showed that the CA09-WT caused the severest lung lesions (3.67 ± 0.33), whereas the CA09 + H3N2-M2 and CA09 + H3N2-M1 only caused moderate lesions (2.67 ± 0.33 and 2.33 ± 0.33, respectively). The IHC assay indicated that the CA09-WT infected lung contained more influenza viral antigens than CA09 + H3N2-M2, and in CA09 + H3N2-M1 infected lungs, the least antigens could be detected (Figure 3E). All the results demonstrated that both M1 and M2 contribute to the virulence of CA09, whereas the CA09 M1 plays a more critical role than M2 in the pathogenicity of CA09 in mice.

### 3.3. CA09 + H3N2-M1 Induced Lower Inflammatory Responses, whereas CA09 + H3N2-M2 Induced Comparable Inflammatory Responses with Wild Virus

**Figure 4:**
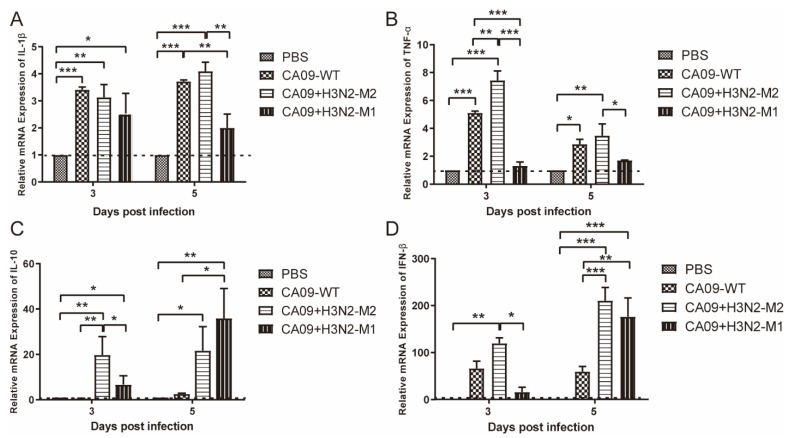
Relative mRNA expression levels of molecules IL-1β (**A**), TNF-α (**B**), IL-10 (**C**), and IFN-β (**D**), in each group in mouse lungs on 3 and 5 dpi. Dashed lines indicate the levels of PBS group. The statistical analysis was performed using GraphPad Prism with two-way ANOVA and one-way ANOVA with post hoc Tukey’s test, and the significance was presented, * *p* < 0.05; ** *p* < 0.01; *** *p* < 0.001.

IL-1β and TNF-α are pro-inflammatory cytokines involved in host immune and inflammatory responses. IL-1β is a key mediator of the inflammatory response. The IL-1β was upregulated in all infection groups on day 3 with similar levels (Figure 4A). On day 5, the levels of IL-1β in the CA09-WT and CA09 + H3N2-M2 groups were significantly higher than in the CA09 + H3N2-M1 group. TNF-α induces diverse cellular response and mediates lung injury during influenza virus infection. The CA09 + H3N2-M1 group induced significantly lower levels of TNF-α on day 3 than the CA09 + H3N2-M2 and CA09-WT groups (Figure 4B). On day 3, the CA09 + H3N2-M2 induced significantly higher levels of TNF-α than the other two infection groups. The results demonstrated that the CA09 + H3N2-M2 induced substantial pro-inflammatory responses with CA09-WT, and the CA09 + H3N2-M1 induced lower levels of pro-inflammatory cytokines.

Pro-inflammatory and anti-inflammatory cytokines are responsible for acute inflammation and minimizing the damage to the host, allowing the clearance of pathogens, and maintaining the balance of host immune response. IL-10 is an important anti-inflammatory cytokine. The IL-10 level in CA09-WT group was similar to PBS group, and lower than CA09 + H3N2-M1 and CA09 + H3N2-M2 groups on both 3 and 5 dpi (Figure 3C). The IL-10 level in CA09-WT group was significantly lower than in the CA09 + H3N2-M1 group on 5 dpi.

IFNs are important in host innate antiviral immune response, and IFN-β is critical in virus controlling [33]. The IFN-β levels in mouse lungs were tested. On 3 dpi, the CA09 + H3N2-M1 induced less IFN-β production compared with the other two viruses (Figure 3D), whereas on 5 dpi, the CA09 + H3N2-M2 and CA09 + H3N2-M1 groups induced significantly higher levels of IFN-β than the CA09-WT group. All the results indicated that low inflammatory response contributes to the attenuation of the CA09 + H3N2-M1, and that M1 and M2 protein may be involved in IFN regulation.

## 4. Discussion

The A(H1N1)pdm09 virus broke out with high mortality and morbidity in the early stage of the 2009 pandemic [42]. The swine influenza viruses containing North American M or Eurasian avian-like M genes are co-circulating in the US swine population [33]. Especially, the Eurasian avian-like M gene has been found becoming endemic in human and swine influenza viruses [43,44]. A study showed that the corporation of Eurasian avian-like NA and M genes is critical for the pathogenicity of A(H1N1)pdm09 [45]. Further studies showed that the Eurasian avian-like M gene has been found critical in virus morphology, replication, transmission, pathogenicity, and host adaptation [21,22,25,46].

M1 and M2 are two major proteins encoded by M segment that have multi-functions during virus replication. However, the effects of M1 and M2 to the viral pathogenicity of A(H1N1)pdm09 have not been evaluated yet. In the present study, both H3N2-M1 and H3N2-M2 decreased viral titers of CA09-WT *in vitro*. Moreover, H3N2-M1 greatly attenuated the virulence of A(H1N1)pdm09 in mice, which suggested that Eurasian avian-like M1 may play a more important role in pathogenicity of A(H1N1)pdm09 in mice than M2. Yang J. et al. showed that M1 of A(H1N1)pdm09 is implicated more than M2 to the viral replication and pathogenicity of chimeric H17 influenza virus [47]. There are discrepancies between *in vitro* and *in vivo* studies that need further elucidation. The final titer of CA09 + H3N2-M2 in viral growth kinetics was significantly attenuated compared with the other viruses *in vitro*. However, the replication of this virus was comparable to CA09-WT, with substantial weight loss in mice. In comparison, the titers of CA09 + H3N2-M1 *in vitro* reached similar levels to CA09-WT at 36 and 48 hpi, yet the pathogenicity is significantly lower in mice, and clinical signs are indistinguisbohable from PBS group. All the data suggested that Eurasian avian-like M1 is critical to the virulence of A(H1N1)pdm09 in mice.

However, the mechanism of Eurasian avian-like M1-affected virulence of A(H1N1)pdm09 is unclear. On one hand, the residues in A(H1N1)pdm09 M1 (30S, 142A, 207N, and 209T) have been proved to be important in virus morphology and transmission [48], whereas the M1 of H3N2 triple reassortant swine virus contain 30D, 142V, 207S, and 209A, which may be critical for the attenuation of CA09 + H3N2-M1 in mice. A residue, 205V, associated with chronic respiratory disease in swine, was also found in A(H1N1)pdm09 M1, but not H3N2-M1 [49]. These genetic determinants in M1 may play important roles in the virulence of A(H1N1)pdm09. On the other hand, the virulence of influenza virus is influenced by cooperation of M1 and M2, and the NA protein. The interaction between M1 and the cytoplasmic tail of M2 at the budding site influenced the assembly of newly synthesized viruses [50]. Whether the cooperation of M1 and M2, or NA influence the pathogenesis by affecting assembly needs further studies.

During influenza virus infection, the virus first invades the epithelial cells along the respiratory tract. Virus RNAs are recognized by pathogen-associated molecular patterns (PAMPs) and damage-associated molecular patterns (DAMPs), triggering natural immune response, and then inducing the secretion of chemokines and cytokines [51,52]. The pro-inflammatory balanced with anti-inflammatory cytokines dictate the ultimate response to initial lung injury by activating local and systemic inflammatory reactions, thereby clearing the infections. In our study, the CA09 + H3N2-M2, still virulent to mice, induced comparable levels of IL-1β and TNF-α compared with CA09-WT, and even induced significant higher levels of TNF-α than CA09-WT on day 3, whereas for anti-inflammatory response, the CA09 + H3N2-M2 induced higher levels of IL-10 than the wild type virus. The CA09 + H3N2-M1 caused less pathological lesions to the lung, and induced lower pro-inflammatory cytokines than the CA09-WT and CA09 + H3N2-M2 on 3 and 5 dpi, and higher levels of anti-inflammatory cytokine than the other two viruses on day 5. The CA09 + H3N2-M1 induced lower IFN-β mRNA levels on 3 dpi, although on day 5, the IFN-β elevated with CA09 + H3N2-M1 was as high as CA09 + H3N2-M2, which may be the result of the replication of CA09 + H3N2-M1 virus from day 3 to day 5. However, as far as we know, M1 or M2 are of not much significance to IFN induction and antagonism. Cao W. et al. found that the variant H3N2 swine influenza virus containing the M segment from A(H1N1)pdm09 induced lower IFN levels, but higher pro-inflammatory cytokine levels [53]. This result is in agreement with our results, which evidences that A(H1N1)pdm09 M is related to lower IFN induction and higher pro-inflammatory cytokine induction, and either the exchange of M1 or M2 resulted in increased IFN and decreased inflammatory cytokines. A study reported that influenza A virus M2 protein is able to regulate mitochondrial antiviral signaling (MAVS)-mediated signaling pathway, which may change the IFN induction level [54]. Moreover, in a study, the NS1 deleted virus was passaged on MDCK cells, and two strains were found to be replicated efficiently due to adaptive mutations on the M segment, which may provide the IFN-antagonizing ability of the M gene with different mechanisms. They [55] reported that the mutations in the M segment changed the expression ratio of M1 to M2, which probably changed the distribution of M1 between the nucleus and the cytoplasm. However, how M1 and M2 influenced the expression of cytokines needs further study.

In conclusion, our study showed that the CA09 M1 protein is more important to the virulence of the CA09 strain. Lower levels of the inflammatory response are mainly responsible for the decreased pathogenicity of CA09 + H3N2-M1. The discrepancy of virus replication ability *in vitro* and in mice may result from the overwhelming innate immune response in mice which clears the viruses. The IFN-β level implied that the M1 protein may be involved in type I IFN response through certain approaches. This study investigated the effect of the M segment by dividing it into two functional parts for the first time. Our results will provide more information on influenza surveillance that will better prevent influenza outbreaks. However, the detailed mechanisms, such as the exact site that is critical to the pathogenicity, need to be further investigated.

## Figures and Tables

**Table 1:** Primer pairs for SYBR green-based real time relative RT-PCR and overlapping PCR.

Genes	Primer Sequences
CA09 M1 + H3N2 M2 inner-F	5′-CTATCAGAAACGAATGGGGGTGCAGATGCAACG-3′
CA09 M1 + H3N2 M2 inner-R	5′-TCACTTGAATCGTTGCATCTGCACCCC-3′
H3N2 M1 + CA09 M2 inner-F	5′-ACCTACCAGAAGCGAATGGGAGTG-3′
H3N2 M1 + CA09 M2 inner-R	5′-TCACTTGAATCGCTGCATCTGCACTC-3′
IL-1β	F: 5′-CACCTGGTACATCAGCACCTCAC-3′
	R: 5′-CATCAGAAACAGTCCAGCCCATAC-3′
IL-10	F: 5′-GGTTGCCAAGCCTTATCGGA-3′
	R: 5′-ACCTGCTCCACTGCCTTGCT-3′
IFN-β	F: 5′-AAGAGTTACACTGCCTTTGCCATC-3′
	R: 5′-CACTGTCTGCTGGTGGAGTTCATC-3′
TNF-α	F: 5′-CGATGAGGTCAATCTGCCCA-3′
	R: 5′-CCAGGTCACTGTCCCAGCATC-3′
GAPDH	F: 5′-CATCACTGCCACCCAGAAGACTG-3′
	R: 5′-ATGCCAGTGAGCTTCCCGTTCAG-3′

## Data Availability

Data sharing not applicable.
